# The stem cell secretome and its role in brain repair

**DOI:** 10.1016/j.biochi.2013.06.020

**Published:** 2013-07-01

**Authors:** Denise Drago, Chiara Cossetti, Nunzio Iraci, Edoardo Gaude, Giovanna Musco, Angela Bachi, Stefano Pluchino

**Affiliations:** a CNS Repair Unit, Institute of Experimental Neurology, Division of Neurosciences, San Raffaele Scientific Institute, 20132 Milan, Italy; b Biomolecular Mass Spectrometry Unit, Division of Genetics and Cell Biology, San Raffaele Scientific Institute, 20132 Milan, Italy; c John van Geest Centre for Brain Repair, Department of Clinical Neurosciences, and National Institute for Health Research (NIHR), Biomedical Research Centre, University of Cambridge, E.D. Adrian Building, Forvie Site, Robinson Way, Cambridge CB2 0PY, UK; d Wellcome Trust-Medical Research Council Stem Cell Institute, University of Cambridge, Cambridge CB2 0PY, UK; e Dulbecco Telethon Institute c/o S. Raffaele Scientific Institute, Biomolecular NMR Laboratory, Center for Translational Genomics and Bioinformatics, Milano, Italy

**Keywords:** Secretome, Mesenchymal stem cells, Neural stem cells, Brain repair, Stem cell transplantation

## Abstract

Compelling evidence exists that non-haematopoietic stem cells, including mesenchymal (MSCs) and neural/progenitor stem cells (NPCs), exert a substantial beneficial and therapeutic effect after transplantation in experimental central nervous system (CNS) disease models through the secretion of immune modulatory or neurotrophic paracrine factors.

This *paracrine hypothesis* has inspired an alternative outlook on the use of stem cells in regenerative neurology. In this paradigm, significant repair of the injured brain may be achieved by injecting the biologics secreted by stem cells (*secretome*), rather than implanting stem cells themselves for direct cell replacement. The *stem cell secretome* (*SCS*) includes cytokines, chemokines and growth factors, and has gained increasing attention in recent years because of its multiple implications for the repair, restoration or regeneration of injured tissues.

Thanks to recent improvements in *SCS* profiling and manipulation, investigators are now inspired to harness the *SCS* as a novel alternative therapeutic option that might ensure more efficient outcomes than current stem cell-based therapies for CNS repair.

This review discusses the most recent identification of MSC- and NPC-secreted factors, including those that are trafficked within extracellular membrane vesicles (EVs), and reflects on their potential effects on brain repair. It also examines some of the most convincing advances in molecular profiling that have enabled mapping of the *SCS*.

## 1. Introduction

Recent advances in stem cell biology hold great promise in the development of non-haematopoietic stem cell-based therapeutics for the treatment of diseases of the central nervous system (CNS), including animal models of multiple sclerosis (MS) [1–3], Alzheimer’s disease (AD) [4], spinal cord injury (SCI) [5] and stroke [6]. Growing evidence suggests that the effects orchestrated by stem cell transplants might not only be associated with the generation of new graft-derived neurons and glial cells [7,8] and that the context in which these cells are injected critically determines some of the outcomes. Thus, cell replacement is not the sole way for transplanted stem cells to foster tissue repair *in vivo* [9]. It is in fact becoming increasingly accepted that stem cells secrete a vast array of proteins – including growth factors, cytokines, chemokines, metabolites and bioactive lipids – that regulate their biology in an autocrine or paracrine manner, while orchestrating multiple interactions with the surrounding microenvironment (*therapeutic plasticity*) [9–11]. This new concept of *stem cell therapeutic plasticity* describes the various therapeutic actions of transplanted stem cells *in vivo* and their capacity to adapt fate and functions to specific microenvironments [12,13].

Among a number of promising stem cell sources, mesenchymal stromal/stem cells (MSCs; also known as *multipotent stromal cells*) and neural stem/precursor cells (NPCs) are being extensively investigated for their capacity to signal to the host upon transplantation in experimental CNS diseases. Following transplants of both MSCs and NPCs, sustained graft-to-host exchanges of signals has lead to trophic effects on endogenous brain cells and beneficial modulatory actions on innate and adaptive immune responses that have ultimately promoted the healing of the injured CNS [2,14,15]. A number of key regulatory pathways have been identified as being shared between MSCs and NPCs, thus suggesting the existence of a stem cell-like *signalling signature* that is likely to be common to other stem/precursor cell types as well [16].

Both targeted/untargeted proteomics and metabolomics are now being extensively applied to identify novel factors of potential therapeutic relevance in the *stem cell secretome* (*SCS*). Moreover, the use of gene expression approaches or culture preconditioning of modified stem cells capable of actively releasing discrete levels of a pro-regenerative secreted factor merits consideration and will be an area of intensive investigation in the near future. Finally, the development of local *vs* systemic stem cell-free therapeutics that use extracellular membrane vesicles (EVs), instead of whole parental stem cells, is emerging as an exciting new concept in regenerative medicine [17].

Here, we have reviewed the current knowledge of the *SCS* from MSCs and NPCs, and examined its potential in brain repair. We have also discussed the on-going main investigative directions aimed at both improving cellular (secretory) activities and characterizing the *SCS* and its regulation in greater detail.

## 2. The *stem cell secretome* and its role in brain repair

### 2.1. Mesenchymal stem cells

MSCs are self-renewing, clonal precursors of non-haema topoietic tissues that were first identified in the bone marrow (BM-MSCs) [18]. Nevertheless, intensive research efforts have suggested alternative tissue sources that include the adipose tissue (ASCs [19]), the dental pulp [20], the placenta [21], the umbilical cord blood (HUCPVCs [22]), the Wharton Jelly (WJSCs [23]), olfactory mucosa [24], deciduous teeth [25], lung and spleen [26], and even the brain [27]. MSCs can be expanded *in vitro* for some time while retaining the potential to differentiate into mesenchymal cell types closely related to the germ layer of origin, such as adipocytes, chondrocytes and osteoblasts [28]. The transplantation of MSCs has emerged as promise for the repair or restoration of several tissues, including the CNS [29]. That MSC transplants possess potential for the treatment of CNS diseases has become clear following the observation of clinical and histological recovery shown in laboratory animals with CNS disease models after the systemic injection of MSCs [30]. However, the mechanisms driving the therapeutic impact of MSC transplants remain unclear. Among a few candidate hypotheses, two main perspectives receiving attention relate to the tissue trophic and immune modulatory effects that transplanted MSCs exert on the host [31,32].

The intracerebroventricular injection of either BM- or ASC-MSCs has been shown to increase lifespan and body weight, ameliorate motor function impairments, and slow the overall deterioration of twitcher mice, as model of Krabbe’s disease (KD), by inhibition of the type of inflammation associated with KD progression [33]. As such, MSC-transplanted twitcher mice showed a significant reduction in cerebral inflammation, including a significant decrease in the numbers of CNS-infiltrating macrophages, and activated microglial cells as compared to sham-treated controls [33]. Other studies also confirmed the immune modulatory properties of MSCs after systemic cell injection in rodents affected by experimental autoimmune encephalomyelitis (EAE), as a model of MS. The systemic injection of both BM-MSCs and ASC-MSCs via immune regulatory and neurotrophic mechanisms [34–36] lead to inhibition of autoreactive T cell responses as well as the stimulation of endogenous oligodendrogenesis [35–38]. Key factors responsible for some of the observed therapeutic effects have been identified as stem cell-secreted hepatocyte growth factor (HGF) [39,40], as well as fibroblast growth factor (FGF)-II, brain-derived neurotrophic factor (BDNF), and platelet-derived growth factor (PDGF)-AB [34]. The effects of both HGF and MSC-CM are mediated through the tyrosine kinase receptor cMet *in vivo*, and have led to enhanced myelin repair and immune modulation, while being fully inhibited by anti-cMet and anti-HGF antibodies [39,40]. Similar anti-inflammatory effects were observed after MSC transplantation in mice with experimental SCI, showing reduction of astro- and micro-gliosis and enhancement of sensorimotor functions [41].

On the other hand, the transplantation of BM-MSCs delivered into the lesion epicentre of rats with experimental SCI reduced the lesion volume and induced axonal regrowth. In this study, the neurite outgrowth was promoted by BM-MSC-secreted BDNF and glial cell line-derived neurotrophic factor (GDNF) [42]. WJ-MSC or ASC-MSCs are also rich in paracrine neuroprotective factors that may account for protective effects *in vivo* after transplantation. The intralesional transplantation of human WJ-MSCs in rats with experimental complete spinal cord transection led to decreased numbers of microglia and reduced astroglial scarring, and was found associated with increased levels of neutrophil-activating protein-2 (NAP-2), neurotrophin-3 (NT-3), FGF-II, glucocorticoid-induced tumour necrosis factor receptor (GITR), and vascular endothelial growth factor receptor (VEGFR)-3 [43].

In a mouse model of Huntington’s disease (HD), intrastriatally transplanted BM-MSCs integrated in the host brain and exerted neurotrophic effects that correlate with increased levels of laminin, von Willebrand factor (VWF), stromal cell-derived factor-1 (SDF-1) α, and the SDF-1 receptor CXCR4, which in turn enhanced angiogenesis in the damaged striatum [44]. The intravenous (i.v.) injection of BM-MSCs in 6-hydroxydopamine (6-OHDA)-induced experimental Parkinson’s disease (PD) exerted anti-apoptotic effects on host dopaminergic (DA) neurons in part via secreted SDF-1 α [45]. Rats receiving MSC transplantation showed significant behavioural recovery both in cylinder test and amphetamine-induced rotation test, compared with controls [45]. Further *in vitro* studies confirmed the relevance of MSC-secreted SDF-1 α in mediating some of the tissue trophic/protective effects of grafted MSCs. As such, MSC-conditioned media (MSC-CM) displayed anti-apoptotic effects on 6-OHDA-exposed PC12 cells *in vitro* and increased dopamine (DA) release from the cells. Incubation with anti-SDF-1α antibodies reduced the anti-apoptotic effects of the MSC-CM and confirmed a key role for MSC-secreted SDF-1 α in the observed neuroprotection. Similarly, intrastriatally transplanted ASC-MSCs protect against 6-OHDA-induced experimental PD in mice [46]. Histological, electrophysiological, neurochemical and gene expression studies suggested that the likely mechanisms by which ASC-MSCs cell grafts rescued the nigrostriatal function involved little direct differentiation of the stem cell graft into functional dopaminergic neurons, rather indirect modulation of the oxidative stress-induced neuroinflammatory environment via the secretion of GDNF, BDNF and nerve growth factor (NGF) at the level of the lesioned substantia nigra [46].

Intravenously injected human MSCs have been found to induce functional amelioration, reduce the infarct volume, and promote neuroprotection in rats with experimental middle cerebral artery occlusion (MCAo), a model of brain stroke. Increased levels of insulin-like growth factor (IGF)-1 as well as induced expression of VEGF, epidermal growth factor (EGF) and FGF-II in the host cells were observed in the brain of transplanted rats, as compared with controls [47]. Interestingly, IGF-1 only was detected *in vivo*, suggesting its direct and specific induction, e.g. by the ischaemic or inflammatory environment, in transplanted MSCs. The intracerebral implantation of WJ-MSCs in experimental brain stroke increased brain plasticity and modulated β1-integrin expression, while also providing exogenous SDF-1α, BDNF and GDNF, which were found in brain areas adjacent to the infarcted tissue [48]. The transplantation of human ASC-MSCs also enhanced angiogenic and neurogenic processes promoted by secreted VEGF, hepatocyte growth factor (HGF), and transforming growth factor (TGF)-β [49,50]. The intracerebroventricular (i.c.v.) injection of ASC-CM reduced the infarct volume and the brain swelling of mice with ischaemia–reperfusion experimental brain injury, by a mechanism of neuroprotection that was dependent on secreted tissue inhibitor of metalloproteinase-1 (TIMP-1) and progranulin [51]. The direct acute (either 1 h or 24 h post-injury) intravenous infusion of ASC-CM induced behavioural and learning recovery of rats with experimental hypoxia–ischaemia (HI) brain injury, while markedly reducing long-term functional cognitive and motor skill impairments by a mechanism regulated by secreted IGF-1 and BDNF [52].

*In vitro* co-cultures with MSCs and SH-SY5Y neuroblastoma cells showed that BM-MSCs promote neuronal/glial survival and neuritogenesis through the secretion of BDNF and β-NGF [53]. Neutralizing anti-BDNF antibodies only partly inhibited these effects, thus suggesting that other factors secreted by MSC also have neuritogenic effects [53].

The characterization of BM-MSC-conditioned media by antibody-based protein array analyses and enzyme-linked immunosorbent assays (ELISAs) showed enrichment of IGF-1, HGF, VEGF and TGF-β, and provided evidence of their role in the increased neuronal survival and outgrowth *in vitro* [54]. Individual trophic factors, or combinations thereof, were however less effective at promoting these effects than whole CM, thus suggesting that the key factors responsible for regulating the MSC secretome remain to be identified [54].

Salgado and colleagues suggested that the *SCS* of human umbilical cord perivascular cells (HUCPVCs) might contain neuro-regulatory factors that increase cell viability and proliferation on primary glial cultures and potentiate the proliferation of neural progenitors as well as neuronal survival/differentiation *in vitro* [55]. In addition to these neuroprotective properties, ASC-MSC-conditioned media also mediated the repair of damaged tissues by inducing neuronal differentiation via NGF-induced activation of 5′ AMP-activated protein kinase (AMPK) in neurogenic PC12 cell line *in vitro* [56]. BM-MSC-secreted factors present in the conditioned medium were also capable of promoting the proliferation, as well as increasing the expression of glial fibrillary acidic protein (GFAP) by neural stem/precursor cells (NPCs) *in vitro*, which suggests an effect towards differentiation into astrocytes [57]. Other evidence implied that MSC-secreted factors might induce oligodendrogenesis upon reducing the generation of astrocytes, and promote oligodendroglial differentiation/maturation of adult NPCs, as shown by treatment of NPCs with rat MSC-CM [58].

Some evidence supports the view that paracrine signalling mediated by MSC-released factors in the *SCS* may also play an essential immune modulatory role in brain regeneration. The immune modulatory capacities of MSCs are suggested by the inhibition of T and B cell proliferation [59,60], inhibition of the production of H_2_O_2_ from neutrophils [61], and T and NK cytotoxicity [62], as well as the differentiation and maturation of monocytes into dendritic cells [63]. Of note, these immune modulatory properties are not only constitutive, but also promoted by proinflammatory cytokines such as interferon (IFN)-γ, tumour necrosis factor (TNF)-α, and interleukins (IL)-1α and -1β that recapitulate *in vitro* the main inflammatory signalling that MSCs are expected to face *in vivo* after transplantation [64].

Several studies reported that the i.v. or intraperitoneal (i.p.) injection of MSCs modulates immune responses *in vivo* to induce peripheral tolerance and ameliorate disease in mice with EAE [65–69]. Zappia et al. showed that i.v.-injected MSCs modulate T-cell responses within the peripheral compartment of EAE mice *in vivo*, and inhibit the proliferation of *ex vivo* derived T cells from MSC EAE-treated mice, upon stimulation with the encephalitogenic *myelin oligodendrocyte glycoprotein* (MOG) *in vitro*. In addition, *in vitro* comparative analyses on tissue specific MSCs such as human BM-MSCs and ASC-MSCs revealed differences that can be relevant to their ability to modulate immune responses after stem cell grafts [63]. Payne et al. provided provocative evidence that distinct immune modulatory and migratory mechanisms might underpin the diverse therapeutic potential of human MSCs from different tissue sources, such as BM-MSCs, ASC-MSCs and WJ-MSCs, *in vitro* and *in vivo* after systemic MSC injection [70]. After having characterized *in vitro* the above MSCs for their immune modulatory effects, these authors assessed their therapeutic efficacy *in vivo* in mice with chronic EAE [70]. *In vitro*, BM-MSCs – but not ASC-MSCs and WJ-MSCs – inhibited T cell proliferation through soluble factors present in CM that were enriched into the inflammatory cytokines TNF-α, IL-6 and IL-10. This secretion profile was not observed with ASC-MSC or WJ-MSC CM, thus suggesting that those soluble factors secreted by BM-MSCs may account for part of their potent *in vitro* immune modulatory effects. Despite this *in vitro* finding, only i.v.-injected ASC-MSCs ameliorated chronic EAE in mice. The disparity between these *in vitro* and *in vivo* findings could then reflect differences in the homing potential of the three tested MSCs types, as implied by differential expression of integrin-α4 and the inability of integrin-α4-negative BM-MSCs to traffic to the CNS [70].

Recently, induced pluripotent stem (iPS) cells have provided an alternative source of functional MSCs for tissue repair [71]. iPSC-derived MSCs were comparable to BM-MSCs in surface marker expression, differentiation potential, and also *in vivo* regenerative potential in the hind limb ischaemia mouse model [72].

Specific roles of secreted factors such as tryptophan catabolites promoted by the activity of indoleamine 2,3-dioxygenase (IDO) [40], IL-6, prostaglandin E2 (PGE2), TGF-β, HGF, leukaemia inhibitory factor (LIF), and human leucocyte antigen molecule 5 (HLA-G5) have been associated with some of the immunosuppressive effects of MSCs [73].

Mouse MSCs co-cultured with splenocytes *in vitro* inhibited T cell proliferation via an IL-6-dependent mechanism [74]. Mouse BM-MSCs also secreted significant amounts of PGE2 *in vitro*, which correlated with the efficacy of EAE inhibition after systemic MSC injection [75]. The immune regulation of transplanted BM-MSCs in EAE required PGE2 and the loss of immune regulatory functions by differentiated MSCs correlated with decreased secretion of PGE2. These findings suggest that PGE2 secretion represents an important mechanism in the control of autoimmune conditions in EAE [75].

Human BM-MSCs express IDO, which catalyses the conversion of tryptophan to kynurenine and exhibits functional IDO activity, resulting in tryptophan depletion and kynurenine production upon stimulation with IFN-γ [76,77]. Co-cultures of MSCs and mixed lymphocyte reactions (MLRs) have been assessed for IDO activity by measuring tryptophan-to-kynurenine conversion in MSC-CM and in MLRs with MSCs. IDO-mediated tryptophan depletion in MSC-CM functions as a T cell-inhibitory effector mechanism in MSC/MLR co-cultures [76]. In addition to tryptophan depletion, catabolites of tryptophan play an active role in dampening the proliferation and inducing the apoptosis of activated allogeneic T cells [78]. Plumas et al. showed that human BM-MSCs induced apoptosis of activated T cells and provided correlative evidence that this took place along with conversion of tryptophan into kynurenine by IDO [77]. In support of this *IDO hypothesis*, another work showed that the oral (or intraperitoneal) administration of synthetic tryptophan metabolites suppressed the proliferation of myelin-specific T cells and reversed paralysis in EAE mice [79].

Moreover, secretion of IL-6 by human BM-MSC triggered a partial inhibition of dendritic cell (DC) differentiation, as shown by a slight but significant decrease in the percentage of mature DCs, when cultured in the presence of IL-6-secreting MSCs [74]. Spaggiari et al. showed that part of the inhibitory effect of human BM-MSCs on DC maturation was mediated by PGE2 [80]. PGE2 directly added to cultures of monocytes blocked their differentiation towards DCs to the same extent as BM-MSCs, and PGE2 inhibitors added to BM-MSC/monocyte co-cultures fully reverted the inhibitory effects of BM-MSCs [80].

Other studies implicate TGF-β and HGF as mediators of the anti-proliferative effect of human BM-MSCs on T cells [81]. This finding has also been corroborated by up-regulation of TGFβ, IL-10, IDO, LIF, and HLA-G transcripts during human MSC/T cell co-cultures [82]. HLA-G alters various immune cell functions, such as NK cell and cytotoxic T lymphocyte-mediated cytolysis [83], allogeneic T-cell proliferation, and dendritic cell maturation [84,85]. Blocking experiments with neutralizing anti-HLA-G antibodies suggested that human BM-MSCs might use secreted HLA-G to suppress T cell proliferation and to inhibit the expansion of CD4^+^/CD25^+^/Foxp3^+^ regulatory T cells, as well as the NK-cell mediated cytolysis and secretion of IFN-γ [86].

More recently, BM-MSC-secreted galectin-1 and semaphorin-3A have been identified as key additional inhibitors of T cell proliferation, while the anti-proliferative effects of MSCs on T cells are completely reverted by blocking antibodies against galectin-1 and semaphorin-3A, or soluble recombinant NP-1, the main receptor of both galectin-1 and semaphorin-3A [87].

Despite all these promising observations, the precise mechanisms and the factors governing the tissue trophic and immune regulatory functions of MSCs are still far from being fully elucidated.

### 2.2. Neural stem/precursor cells

Multipotent neural stem/precursor cells (NPCs), supporting self-renewal and differentiation into neurons, astrocytes, and oligodendrocytes, reside within specialized niches such as the sub-ventricular zone (SVZ) of the lateral ventricles and the subgranular zone (SGZ) in the dentate gyrus of the hippocampus in the adult mammalian CNS [88]. NPCs hold great potential for the treatment of neurological disorders, not only because they may provide a (tissue-specific) cellular reservoir for the replacement of lost or damaged cells, but also because of several bystander capacities, such as tissue trophic support and immune regulation, that have been described *in vitro* and *in vivo* following NPC transplants [89].

Neurotrophic growth factors like NGF, BDNF, ciliary neurotrophic factor (CNTF) and GDNF have been found to be increased after NPC transplantation and are likely responsible for the prevention of neuronal programmed cell death and glial scar formation in both primary and secondary neurodegenerative disorders – such as SCI, ischaemic stroke, HD, PD, and demyelinating disorders[6,90–95]. NPCs constitutively produce and secrete such neurotrophic factors which promote the growth of host spinal axons after brain injuries. In addition, augmentation of natural growth factor production by transduction with NT-3, also secreted by NPCs, extends the spectrum of host axon sensitivity to NPC grafts [92]. Interestingly, over-expression of NT-3 also resulted in a collateral decrease in the expression of other neurotrophins, thus suggesting the existence of a complex reciprocal interplay between different growth factor signal transduction pathways in NPCs [92]. In ischaemic stroke *in vivo*, transplanted NPCs secreted tissue trophic factors such as VEGF, but also induced in the host the re-expression of guidance molecules (e.g. slit/Robo signalling and thrombo-spondins 1 and 2), through which NPCs regulate dendritic sprouting, axonal plasticity and axonal transport *in vitro* [96]. Likewise, striatal neuronal protection and enhanced motor function in HD rats showing poor integration of grafted NPCs, but high levels of NPC-secreted BDNF, both *in vitro* and *in vivo*, suggested that the neuroprotective effects of transplanted NPCs work partly through BDNF secretion [94]. The intrastriatal NPC transplantation in 6-OHDA-induced experimental PD rats induced behavioural recovery and protected from dopaminergic depletion by stem cells factor (SCF)-mediated anti-apoptotic effects [97]. Mouse NPCs unilaterally grafted into the midbrain of aged mice, which contains a large population of dysfunctional but living dopaminergic neurons, displayed a strong tropism for tissue lesions and migrated towards these critical sites to release GDNF, which prevent cell death and facilitate the regeneration of targeted cell populations [98]. The transplantation of Lewis X (LeX) and CXCR4 (Le^+^/CX^+^) NPCs into superoxide dismutase (SOD)1-G93A transgenic mice, as a model of amyotrophic lateral sclerosis (ALS), led to neuroprotection that correlated with increased spinal cord levels of VEGF and IGF-1 [99]. Systemically injected (either intravenously or intrathecally) NPCs first promoted endogenous remyelination in mice with chronic EAE [100]. More recently, EAE studies with i.c.-injected NPCs have revealed that NPC-secreted PDGF-AA and FGF-II are responsible for the activation of the proliferation of host oligodendrocyte progenitor cells (OPCs) and the promotion of their maturation into remyelinating oligodendrocytes [101].

Madhavan et al. have examined the dynamics of the cross-talk between NPCs and their cellular environment using co-cultures with primary neural cells that are exposed to 3-nitropropionic acid (3-NP), an inhibitor of the mitochondrial respiratory complex II, as an *in vitro* model of HD-like oxidative stress. Enhanced NPC secretion of BDNF, CNTF, and VEGF was assessed on cell culture supernatants and was shown to stimulate a robust SOD 2-mediated anti-apoptotic and antioxidant effect on neurons [102]. The neuroprotective effects of NPC-CM were also confirmed by experiments carried out in another *in vitro* model of HD with cerebral hybrid neurons (A1) transfected with either wild type or mutant Huntingtin [103]. When mutant Huntingtin-transfected A1 cells were exposed to NPC-CM, a significant reduction of Huntingtin aggregates and levels of N-terminal proteolytic cleavages of mutant Huntingtin were observed [103]. NPC-CM also reduced the extent of N-methyl-d-aspartate (NMDA)-induced excitotoxic cell death in hippocampal slice cultures. Mass spectrometry (MS) analyses on NPC-CM identified the novel neuroprotective peptide pentitin, a putative breakdown product of the insulin B chain [104]. Pentitin prevented NMDA-mediated toxicity of both mature and immature neurons, thus confirming a role of this secreted factor in part of the neuroprotection observed with NPC-CM [104]. Human NPCs transplanted in the rat cortex 1 week post-stroke successfully engrafted and migrated towards the lesion. Interestingly, the functional recovery correlated well with increased vessel formation and enhanced BBB integrity as well as suppression of inflammation. VEGF was identified as the main NPC-secreted factor responsible for some of the effects *in vivo*, and pre-treatment of grafted human NPCs with the humanized anti-VEGF-A monoclonal antibody *Bevacizumab* significantly reduced the NPC-mediated neovascularization in the peri-infarct cortex [105]. Moreover, NPC-grafted rats showed significantly fewer Iba1-positive monocytes/macrophages in the peri-infarct cortex, compared with sham-treated controls. Again, this anti-inflammatory effect was completely neutralized by *Bevacizumab* treatment, thus implying that NPC-secreted VEGF was important for both pro-angiogenic and immune regulatory effects [105].

Transplanted NPCs also show remarkable immune regulatory properties *in vitro* and also *in vivo* after transplantation into animal models of MS, SCI and stroke [3,7,106]. However, only a few reports have shown neat evidence of the effects of grafted NPCs on the host immune system. Injecting NPCs subcutaneously (s.c.) into mice with relapsing EAE, rather than intravenously, has turned out to be a smart approach to have transplanted NPCs predominantly target secondary lymphoid organs – where their interactions with the host immune cells can be studied – with a negligible accumulation into the CNS. In fact, s.c.-injected NPCs accumulate and survive over 2 months within draining lymph nodes, where a focal up-regulation of major developmental stem cell regulators, such as bone morphogenetic protein (BMP)-4, Noggin and Sonic hedgehog are observed [3]. *Ex vivo* studies showed a significant down-regulation of the co-stimulatory molecules CD80/B7.1 and CD86/B7.2 on DCs extracted from the lymph nodes of NPC-injected EAE mice, and this finding was also confirmed within *in vitro* NPC/DC co-cultures. Further *in vitro* experiments identified a novel, highly NPC-specific, BMP-4-dependent mechanism hindering DC maturation that was advocated as crucial. The evidence that BMP-4 plays a central role in DC maturation was provided by using BMP antagonist Noggin, which reverted the antigen presentation capacity of naive PLP-pulsed DCs co-cultured with NPCs *in vitro*. This was the first identification of a member of the TGF-β/BMP family of stem cell developmental regulators as a novel tolerogenic factor released by immune regulatory NPCs. A more recent study has shown that the intravenous administration of NPCs ameliorates EAE by selectively inhibiting the differentiation of encephalitogenic T helper 17 (Th17) cells through secreted factors. Cao et al. also identified LIF as the key factor responsible for the observed inhibition of Th17 cell differentiation mediating the efficacy of NPCs in EAE, and elucidated the signalling pathway behind this novel mechanism of action, where LIF antagonizes interleukin (IL)-6-induced Th17 cell differentiation through Extracellular signal-regulated kinase (ERK) and Suppressor of cytokine signalling (SOCS)3-dependent inhibition of Signal transducer and activator of transcription (STAT)3 phosphorylation [107].

High levels of nitric oxide (NO) and PGE2 were detected in the supernatants of NPCs co-cultured with T cells. Both NO and PGE2 were induced in T cells co-cultured with NPCs, compared with T cells only or NPC-only control cultures, thus suggesting that interactions with T cells might promote or enhance NO and PGE2 production from NPCs [108]. Moreover, inducible NOS (iNOS) and microsomal type 1 PGE synthase (mPGES-1) was detected only in NPCs co-cultured with T cells, reinforcing the view that NO and PGE2 production in NPCs is induced by activated T cells. To determine whether NO and PGE2 were involved in T cell suppression by NPCs, specific inhibitors for NO and PGE2 production were investigated and found to restore T cell proliferation [108].

NPC-secreted heme oxygenase-1 (HO-1) has also been implicated in T cell suppression, as shown by partial loss of immune suppressive (e.g. anti-proliferative) effects in NPCs pre-treated with the competitive inhibitor of HO-1 protoporphyrin IX (SnPP) prior to being co-cultured with T cells [109].

All these studies confirm the importance of the interactions between NPCs and immune cells to reconfigure the deleterious inflammatory environment and thus promote the healing or regeneration processes. While the description of the processes involved in NPC protection is still a matter of intense investigation, the ability of NPCs to secrete neuroprotective and immune modulatory factors is remarkable, and indicates that there is a lot more to learn about functional stem cell plasticity.

## 3. Extracellular membrane vesicles as specialized *cargoes* of the *stem cell secretome*

In addition to paracrine soluble factors that stem cells release at the level of the extracellular space, intensive research efforts are being devoted to investigating the role of secreted EVs in the therapeutic potential of stem cells [110,111]. EVs can be considered as paracrine or endocrine signalling vehicles, given that they might transport defined signalling molecules such as cytokines to target cells at distant sites [112–114]. To date, the biochemical composition, the complex biogenesis of these vesicles, and their physiological role have only partially been elucidated. Despite the lack of detailed characterization, their potential as mediators of cell communication has not gone unnoticed, since these vesicles have remarkable features, including the ability to transfer proteins and functional genetic materials such as micro RNAs (miRNAs) to other cells [115–119]. It is also worth noting the bidirectional exchange of information between stem cells and target cells with EVs released from stem cells that may confer a stem-cell-like phenotype to injured cells, leading to the activation of self-regenerative programs [110]. On the other hand, EV-mediated transfer of tissue-specific information from injured (host) cells to grafted stem cells may reprogram the latter to gain phenotypic and functional characteristics of the cell of origin. It would seem likely that the potential of meaningful repair of the brain after injury will depend on the bi-directionality of this communication [9]. To date, only a few studies have been conducted that have characterized the protein composition of MSC-derived EVs in more detail [120], with the genetic cargo of EVs being the most thoroughly analysed component [116].

Liquid chromatography (LC)/MS–MS analysis of the MSC-EVs proteome identified 730 proteins, with features common with EVs derived from other types of cells as well as some distinct to MSC-EVs. The MSC-EVs proteome integration with MSC self-renewal and differentiation-related genes, and the proteome of MSC-conditioned media, provides a list of candidate molecules and their associated pathways through which MSC-EVs can promote the therapeutic effects including surface receptors like platelet-derived growth factor receptor (PDGFRB); signalling molecules like mitogen-activated protein kinase 1 (MAPK1); cell adhesion mediators like fibronectin 1 (FN1); and MSC-associated antigens. The MSC-EV proteome was also profiled by MS and antibody array by Lai et al., who succeeded in identifying 857 proteins [119]. A predominant feature of the MSC-EV proteome was the presence of all α and β chains of the 20S proteasome and the three beta subunits of the immunoproteasome, which correlated with a significant reduction of the oligomerized protein *in vivo* in mice with myocardial infarction treated with MSC-EVs [119,121].

Katsuda et al. proposed the use of adipose tissue-derived MSC-EVs as a promising approach to delivering the most important β amyloid (Aβ)-degrading enzyme neprilysin (NEP) peripherally and/or directly into the brain in AD [122]. Immunoblot analyses revealed that exosomes (a class of EVs) secreted by ASC-MSCs contain NEP and the enzyme activity assay confirmed that these exosomes exhibit NEP-specific activity only in ASC-MSC-derived exosomes, but not in BM-MSC exosomes. ASC-MSC-derived exosomes were transferred into N2a cells overproducing Aβ as shown by co-cultures of PKH26-labelled ASC-MSCs with PKH67-labelled N2a cells, and were suggested to decrease both secreted and intracellular Aβ levels. This was the first report of the isolation of exosomes containing enzymatically active NEP from cultured cells, suggesting a promising new approach for Alzheimer’s disease (AD) treatment.

EVs derived from MSCs expressed regulatory molecules such as programmed death ligand-1 (PD-L1), galectin-1 and membrane-bound TGF-β as detected by flow cytometry [123]. Based on these findings, *ex vivo* immune modulatory effects of MSC-derived EVs on antigen-activated EAE mice lymphocytes were investigated. Results showed that EVs effectively inhibit auto-reactive lymphocyte activation and proliferation, and promote the secretion of lymphocyte-derived anti-inflammatory cytokines like IL-10 and TGF-β. This provides evidence that MSC-derived EVs serve as vehicles for several MSC-specific tolerogenic molecules. Besides the involvement of MSC-EVs in the induction of immune tolerance, the evidence that the immune modulatory effects of NPCs might also be partially mediated by EVs arises from *in vitro* experiments in which the culture supernatants of NPCs suppress the activation and proliferation of T cells by apoptosis and the cell cycle arrest. Isolated EVs added to the supernatant of cultured T cells result in a similar suppression by G0/G1 cell cycle arrest [124].

In addition to the release of soluble factors, the beneficial effects observed following stem cell therapies in regenerative medicine could rely on the secretion of pro-regenerative signals delivered by EVs. Systemically MSC-secreted EVs were found to have the same beneficial effect in protecting against ischaemia–reperfusion-induced acute and chronic kidney injury by inhibiting apoptosis-stimulating tubular epithelial cell proliferation and significantly reducing the impairment of renal function [125]. In this experimental model, the effect of EV injection was comparable to that observed with MSCs, indicating that EVs mimic a substantial part of the beneficial effects of the cells of origin. This in turn suggests that EVs mediate, at least partly, the MSC-regenerative potential, opening up new potential applications of EVs as *specialized secretome cargoes*.

While the beneficial and protective effects of EVs secreted by MSCs in tissue repair have been reported in heart [121] and kidney pathological conditions, their use for brain repair is still not well characterized in *in vivo* models. Nevertheless, as a bi-lipid membrane vesicle with many membrane-bound proteins and a diverse cargo, EVs may represent an ideal therapeutic agent that has the potential to home to target tissues and treat neurological diseases. With that aim in mind, a more in-depth characterization of the neurotrophic and regenerative factors present in the EV cargoes secreted by both MSCs and NPCs is mandatory in the attempt to select and identify the molecules responsible for the therapeutic effects of the *SCS*.

## 4. Investigative directions in characterizing the stem cell secretome

The new emerging view – that is, to foster the repair of an injured tissue by harnessing the paracrine factors instead of using whole cells – introduces a radically different dimension to the therapeutic applications of MSCs and NPCs in regenerative medicine. This has alerted the scientific community to the challenging investigation of the secreted molecules in the *SCS*, be they soluble or delivered by EVs, which may be responsible for some of the beneficial effects observed after stem cell transplantation.

These efforts have been greatly facilitated by significant technological advances in the field of proteomics during the last two decades [126]. Despite this potential, the knowledge of the secreted proteins in mediating MSCs and NPCs function is still very limited. The most commonly used approaches to identifying secreted proteins include gel-based (2-D or 2-D Fluorescence Difference Gel Electrophoresis (DIGE)) or gel-independent (1-D or 2-D LC–MS/MS) techniques [127]. The highly bioactive molecules secreted in the SCS are often difficult to identify using conventional gel-based techniques; LC–MS/MS is currently the preferred approach, often employed in conjunction with isobaric quantification with stable isotope labelling with amino acids in cell culture (SILAC), isobaric tag for relative and absolute quantitation (iTRAQ) or isotope-coded affinity tag (ICAT) labelling. With this aim, multiplex antibody-based techniques offer high sensitivity (typically 1–10 pg/ml) as well as high specificity, reproducibility across a broad range of concentrations, and the potential for massively parallel experiments. This multiplexing methodology can be used on planar microspot array (e.g. RayBio® cytokine arrays) or bead-based assays (e.g. Luminex® xMAP technology). The limitation of the targeted antibody-based methods lies in the pre-selection of the analytes included in the assay and the exclusion in the assay panel of those molecules with specific antibodies not commercially available. On the other hand, an untargeted proteomic approach will allow a huge range of molecules to be covered, the most intriguing aspect being to discover new, undefined secreted factors that are therapeutically relevant to the *SCS*.

Mouse and human MSC *SCS* have been recently characterized using mostly LC–MS/MS approaches [128–134]. Sze et al. have provided the whole spectrum of secretory proteome of human embryonic stem (ES) cell-derived MSCs, which was consistent with the reported paracrine effects of whole (parental) MSCs on different cellular systems and diseases [135]. By using a combination of two techniques – LC–MS/MS and antibody arrays – they suggested a molecular basis for the use of MSC-CM in modulating repair after injuries. Although shotgun proteomics analysis by LC–MS/MS is a sensitive technique and has high throughput capability, it is difficult to detect small proteins/peptides, which include most of the cytokines, chemokines, and growth factors. This was partially overcome by the use of antibody arrays (RayBio® cytokine arrays) increasing the reproducibility of the proteomic profiling of the MSC secretion *in vitro*. The resulting proteomic profile of secretion by MSCs included almost all the cytokines and chemokines that were previously reported [132] to be secreted by MSCs, such as epithelial-derived neutrophil-activating peptide 78 (ENA78), growth factors (VEGF, HGF), growth factor-binding proteins such as insulin-like growth factor binding protein 2 (IGFBP2), cytokines (IL-1 β, IL-6, IL-8, TGF-β) and tissue metalloproteinase inhibitors (TIMP) 1 and 2. From the total number of 201 unique proteins (132 using LC–MS/MS and 72 using antibody array) identified in the cultured medium, more than 85% were validated at the mRNA level by cDNA microarrays or quantitative RT-PCR. In addition, computational analyses were used to predict the roles of the *SCS* components in metabolism, angiogenesis, immune response and differentiation, providing molecular support for an MSC-mediated paracrine effect on tissue repair and regeneration in MSC transplantation studies [132].

To characterize the MSC *SCS*, Sarojini et al. applied the LC–MS/MS proteomic approach to MSC-CM and identified 19 secreted proteins including extracellular matrix structural proteins, collagen processing enzymes, pigment epithelium-derived factor (PEDF) and cystatin C (Cys C). MSC *SCS* has been shown to induce the chemotactic response of human fibroblasts, with PEDF being the most abundant protein in the *SCS* and the predominant fibroblast chemoattractant in the conditioned medium evaluated by immunodepletion and reconstitution experiments [133]. Later, the same authors, by using LC–MS/MS, identified cysteine-rich protein 61 (Cyr61) in the MSC *SCS* that contributes to the promotion of angiogenesis, as previously reported [129]. By using angiogenesis assays, they demonstrated that the MSC’s *SCS* promotes the morphogenesis of endothelial cells in a dose-dependent manner *in vitro*, as well as neovascularization *in vivo*. In addition, the immunodepletion of Cyr61 abrogates the pro-angiogenic effect of MSC *SCS* both *in vitro* and *in vivo*. Finally, the addition of human recombinant Cyr61 to the depleted *SCS* restores its pro-angiogenic effects, thus confirming that Cyr61 is one of the key factors in the MSC *SCS* promotion of vasculature remodelling. Collectively, these data suggest that MSCs secrete a panel of functionally active factors that act in the healing of the injured tissue microenvironment. MSC-secreted chemoattractants, such as PEDF, eventually stimulate the migration of fibroblasts that is important for scaffold building, while the secretion of Cyr61 promotes neovascularization [129].

Mouse bone marrow stromal cell-derived NPCs provide a protective function by secreting prosaposin (PSAP), a protein that is capable of rescuing mature neurons from apoptotic death. This factor is identified as the lead protein in the *SCS* of NPCs by tandem mass spectroscopic profiling, and further validated by western blotting and immunocytochemistry [131]. In this study, the NPC *SCS* protected from apoptotic death induced by 6-OHDA in cultures of rat PC12 neuronal cells, human ReNcell CX cell-developed neuronal cultures and rat cortical primary neurons. The proof that PSAP is a key factor in this protection was achieved by using *SCS* immunodepleted of PSAP that lacked the anti-apoptotic effect of *SCS*, as well as by adding the recombinant peptide of PSAP in the NPC *SCS* that prevented cell death [131]. Aside from PSAP, six other secreted proteins were detected in the NPC *SCS* by LC–MS/MS analysis, such as collagen α-2(I) chain (COL1A2), fibronectin (FN), granulin (GRN), Cys C, neurotensin (NT)/neuromedin N (NN) and secreted protein acidic and rich in cysteine that can be further investigated for the anti-apoptotic activity in mature neurons [131].

Dahl et al. were the first to combine two-dimensional gel electrophoresis (2-DE) and mass spectrometry to characterize the secreted factors in conditioned media of rat neural hippocampal progenitor cells [136], confirming the practicality of proteomic analysis to decipher the molecular aspect of *SCS*. The authors succeeded in the identification of a number of stem cell factors in NPC-CM, which included Rho-guanine nucleotide dissociation inhibitor 1 (RhoGDI), phosphatidylethanolamine binding protein (PEBP), polyubiquitin, immunophilin FK506 binding protein 12 (FKBP12) and Cys C [136].

Several other studies identified major NPC intracellular proteins significantly regulated during neural stem cell differentiation using mostly 2-DE and LC–MS/MS approaches [137], most of them are actually focused on the proteome of cell lysates rather than the characterization of the *SCS* itself except for proteomic comparative studies of proteins secreted from neurons, astrocytes and the rat adult hippocampal nerve–glial precursor cell line [138]. In addition to adhesion and antioxidant proteins, including glutathione S-transferases and peroxiredoxins, this study showed that the composition of the NPC-secreted proteins contains some molecules, such as protein molecular chaperones, proteasome subunits, and metabolic enzymes that lead to the identification of new roles for the extracellular space within the CNS. The most unexpected groups of proteins found outside the cell are those involved in carbohydrate metabolism, such as lactate dehydrogenase, creatine kinase, and malate dehydrogenase [138]. Moreover, this comparative study showed that the repertoire of secreted proteins is unique for each cell type and represents a distinct set of molecular identifiers for individual phenotypes within the CNS.

Roche et al. provided a comparative profiling of the SCS of human ES cells, MSCs and marrow isolated adult multilineage inducible (MIAMI) cells to address the molecular and cellular basis of their regenerative potential [126]. This proteomic screening establishes the closer relationship between MSCs and MIAMI cells, while ES cells were more divergent. Proteins characteristic of MSCs include transgelin-2 (TAGLN2), phosphatidylethanolamine-binding protein 1 (PEBP1), heat-shock 20 kDa protein (HSP20/HSPβ6), and programmed cell death 6-interacting protein (PDC6I), among others. These comparative studies are very useful in providing a general *SCS* overview, since the poly-pharmacies secreted by MSCs or NPCs are potentially more important than any single factor in stimulating CNS repair.

Nonetheless, the key (still unsolved) challenges in *SCS* analysis include the interference of serum proteins in the pure cell population from the donor tissue that contain many overlapping components and can interfere with the detection.

To overcome this problem, cells are often cultured in serum-free media or media with defined components including eventually the analysis of unconditioned medium to identify possible serum contaminants. Remarkably, a number of multiplex methods (RayBio® cytokine arrays or Luminex® xMAP technology) have been developed, allowing the detection and quantification of up to several hundred analytes in a single sample simultaneously, even in the presence of serum, thanks to the calibration standards diluted in unconditioned medium [139]. Another solution to avoid the background of serum-containing media has been developed exploiting the combination of click chemistry and pulsed SILAC to selectively enrich and quantify secreted proteins [140]. This study emphasises the need for careful evaluation of *SCS* profiling, since serum starvation has a strong impact on the secretion of many biologically important proteins, such as cytokines and growth factors. In addition, the combination of in-depth proteome profiling and the ability to resolve protein secretion over very short time spans will be especially helpful in investigating the regenerative properties of the *SCS*.

Therefore, better characterization of the *SCS*, challenging the discovery of new potential regenerative factors, will be achieved by the application of unbiased global discovery techniques, such as LC/MS and the correlation of the resulting data with those obtained by complementary approaches, such as ELISA- or Luminex®-based assays [141]

Moreover, based on the detection of metabolic enzymes in the extracellular space within the CNS and secreted by the rat adult hippocampal nerve–glial precursor cell line [138], the metabolomics of the *SCS* would be promising in improving the knowledge of the metabolic processes accompanying and underpinning stem cell-secreted signalling. In contrast to classical biochemical approaches that often focus on single metabolites, metabolomics is emerging as a powerful tool in CNS research involving the collection of quantitative data on a broad series of metabolites in an attempt to gain an overall understanding of metabolism and/or metabolic dynamics associated with conditions of interest [142]. All these techniques are complementary, and the combination of protein abundance data generated via a proteomic approach and the metabolic data from a metabolomic study should both facilitate the global comprehension of changes in *SCS* profiles under different conditions in regenerative medicine.

The first attempt at applying metabolomic profiling to *SCS* characterization has been carried out by Cezar and co-workers, who measured and identified small molecules secreted by human ES cells and ES cell-derived NPCs in response to valproate for developmental toxicity testing [143]. To date, metabolomic reports on the *SCS* in CNS regenerative medicine are still not available, except for a MSC SCS metabolomic characterization in the context of biomaterials designed for orthopaedic application [144]. Three case studies were reported to illustrate the application of metabolomics to the study of MSC responses to titania-nanopillared substrata. The results highlighted that l-ornithine was a key metabolite significantly modulated in abundance among different heights of substrata. Therefore, increased understanding of the metabolites that become depleted or that accumulate in the medium during the acquisition of a specific stem cell fate might provide a minimally invasive means of assessing the extent of cell differentiation, the SCS being regulated in the microenvironment and, and of predicting the likely changes that the host would make in response to stem cell- or stem cell free-based regenerative strategies.

## 5. Investigative directions to improve the stem cell secretory activities

The current approaches to increasing the production of desired trophic and immune regulatory factors, thus augmenting stem cell paracrine effects, involves (i) different preconditioning strategies that include hypoxic exposure, exposure to cytokine cocktails [14] and preconditioning through cell–cell interactions, or alternatively (ii) gene expression approaches to stem cell engineering.

### 5.1. Stem cell preconditioning

MSCs placed under serum-deprived conditions mimicking an *in vitro* model of ischaemia and hypoxic preconditioning, secreted increased levels of pro-survival and pro-angiogenic autocrine, paracrine, or juxtacrine factors that include VEGF-A, angiopoietins (ANGPTs), IGF-1, and HGF [145]. The mechanism of this hypoxia-induced preconditioning correlated with the induction of the hypoxia-inducible factor (HIF), a transcription factor that binds to the hypoxia response elements in a number of HIF target genes, leading to hypoxia-induced protection against brain ischaemia[146].

Rats with experimental traumatic brain injury (TBI) infused through the tail vein with human MSC-CM collected from hypoxic *in vitro* preconditioning performed significantly better in both motor and cognitive function tests and showed increased neurogenesis and decreased brain damage, compared with TBI rats treated with MSC-CM collected from normoxic *in vitro* pre-conditioning [147]. *In vitro* hypoxic preconditioning was found to enhance the secretion of bioactive factors by MSCs, including HGF and VEGF [147]. Moreover, pro-inflammatory cytokines such as IFN-γ, TNF-α and IL-1α or IL-1β were required to induce immunosuppression by MSCs through the concerted action of chemokines and NO [148]. To determine whether cytokines secreted by activated T cells induce immunosuppression by MSCs the authors used neutralizing antibodies against various cytokines in the anti-CD3-activated splenocyte supernatant (SupCD3-act), before its addition to co-cultures with MSCs. The treatment with anti-IFN-γ antibodies completely restored the proliferation of T cell blasts co-cultured with MSCs supplemented with SupCD3-act, thus confirming the indispensable function of soluble IFN-γ in T cell proliferation. Moreover, the simultaneous neutralization of TNF-α and IL-1α and IL-1β in SupCD3-act before its addition to the MSC-T cell co-cultures completely reversed the inhibition of T cell proliferation, while the antibodies had no effect, neither individually nor in pairs. Therefore, IFN-γ synergized with TNF-α and IL-1α or IL-1β to promote the anti-proliferative effects of MSCs on T cells [148]. Furthermore, MSCs from mice deficient in IFN-γ receptor 1 (IFN-γR1^−/−^) were incapable of suppressing anti-CD3-induced splenocyte proliferation in co-cultures [148].

MSCs responded to IFN-γ in a dose-dependent manner *in vivo*, with increased concentrations of IFN-γ being more efficient at eliciting higher MSC-mediated immune regulatory effects [149]. Several *in vitro* and *in vivo* data are available supporting the role of MSC licensing in the induction of measurable and effective immune regulation [150].

Since pro-inflammatory cytokines have been shown to be essential for the activation of some stem cell-dependent functional effects, it can be reasoned that preconditioning stem cells with cytokines *in vitro* (e.g. prior to cell transplantation) would augment their modulation of the host immune response through paracrine factors. In the attempt to exploit the synergic effects, a cocktail of cytokines, conditioned medium or serum has been employed to stimulate MSCs *in vitro*. Incubation of MSCs with inflammatory-like serum harvested from LPS-stimulated rats induced reactive secretion of a soluble form of TNF receptor 1 (sTNFR1) *in vitro* [151]. The reactivity of this secretion was confirmed by the activation of the TNFR1 downstream nuclear factor kappa-light-chain-enhancer of activated B cells (NFκB) complex signalling pathway in target cell lines that express green fluorescent protein (GFP) under the control of the NFκB promoter. The contribution of NFκB complex to the reactive secretion of sTNFR1 from MSCs was demonstrated in alteration of the protein secretion. Moreover, the blockage of the reactive secretion of sTNFR1 when using a specific inhibitor peptide of NFκB suggested once again that the anti-inflammatory ability of MSC is not innate, but is rather induced by the surrounding inflammatory cytokines. The reactive secretion of sTNFR1 by MSCs was also observed after treatment with neutralizing anti-sTNFR1 antibodies, suggesting that NFκB activation in MSCs is regulated by multiple, not necessarily redundant, functional cytokine receptors [151].

*In vitro* preconditioning with IL-6 enhances the therapeutic effects of the transplantation of NPCs in experimental ischaemic stroke in mice [152]. Syngeneic NPCs harvested from the foetal SVZ were preconditioned with IL-6 *in vitro* and then intracerebrally transplanted 6 h or 7 days after transient MCAo. IL-6 pre-conditioning protected the grafted neural stem cells from ischaemic reperfusion injury through up regulation of manganese SOD2, a primary mitochondrial antioxidant enzyme, and induced secretion of VEGF with the resulting promotion of angiogenesis. Furthermore, transplantation of IL-6-preconditioned NPCs significantly attenuated infarct size and improved neurological performance, compared with MCAo mice transplanted with non-preconditioned NPCs. By taking advantage of RNA interference against STAT3 on NPCs before transplantation, the authors also demonstrated that this IL-6-induced amelioration of ischaemic insults was due to the activation of STAT3 [152].

A better understanding of how the cytokines expressed in the CNS inflammatory or ischaemic environment *in vivo* modulate the therapeutic effects of MSCs or NPCs is therefore a prerequisite to developing more effective protein-based pre-conditioning approaches.

Promotion of cell-to-cell interactions within the heterogeneous stem cell population itself has a profound impact on the *SCS* [153]. *In vitro* 3D culture methodologies facilitate greater cell-to-cell contact and interactions of cells with the extracellular matrix (ECM), allowing cells to adapt to their native morphology, which may affect this signalling activity [154]. As such, aggregation of human MSCs in culture with 3D spheroids provides an effective procedure in prompting the MSCS to secrete tumour necrosis factor-inducible gene 6 protein (TSG-6), and thereby, enhance their anti-inflammatory effects [153]. The spheroid-human MSCs hybrid aggregates also secrete high levels of stanniocalcin-1 (STC-1), a protein with both anti-inflammatory and anti-apoptotic properties. The increased secretion of TSG-6 and STC-1 is also consistent with the enhanced suppression of inflammatory responses in spheroid-human MSCs hybrid/LPS-activated macrophage co-cultures [153].

This mechanical pre-conditioning could offer less control for the *SCS* components than other physiological and molecular approaches like hypoxia or cytokine preconditioning. On the other hand, the impact of these later pre-treatments is transient owing to the self-regulatory mechanisms. In this line, stem cell engineering has the advantage of achieving sustained responses.

### 5.2. Stem cell engineering

Stem cells have been genetically engineered with a number of transgenes to more effectively secrete increased amounts of (tissue) trophic factors. Several approaches to genetically modifying stem cells, including viral and non-viral delivery of technologies, have been used to force/increase the secretion of soluble proteins and to control the *SCS in vivo*, after transplantation [14].

After the transplantation of genetically engineered MSCs as the delivery vehicles of BDNF, NGF or VEGF, significant behavioural recovery and reduction of striatal degeneration was observed in mice with experimental HD [155–157].

The intracerebral transplantation of MSCs modified to overexpress BDNF was also significantly more efficacious in eliciting functional recovery in rats with experimental brain ischaemia, many of the improvements correlating well with the presence of fewer apoptotic cells in the ischaemic boundary zone *in vivo* [158]. Early (24 h) intracerebral transplantation of MSCs modified to overexpress VEGF also improved neurological deficits and reduced infarction volume in MCAo rats [159].

The intrastriatal injection of human umbilical cord mesenchymal stem cells (HUMSC) modified to overexpress VEGF led to a significant reduction of apomorphine-induced rotations, and a revival of tyrosine hydroxylase (TH) immunoreactivity at the level of the lesioned striatum and substantia nigra in rotenone-induced experimental chronic PD rats [160]. A key finding of this study was that VEGF overexpression not only protected host DA neurons, but also increased the levels of endogenous VEGF in the striatum, and promoted the direct differentiation of grafted HUMSCs into TH-positive neurons *in vivo* [160].

Overexpression of GDNF in human NPCs provided protection from nitroprusside-induced apoptosis *in vitro*; while the transplantation of xenogeneic NPC-GDNF directly into the brain of TBI rats showed much higher survival, migratory capacities towards the lesion and neurogenic potential, and led to better cognitive functions as early as 6 weeks after transplantation, compared with TBI rats transplanted with untransduced control NPCs [161].

NPC-VEGF showed increased survival, and provided higher neuroprotection, neoangiogenesis, protection from apoptotic cell death and functional recovery after transplantation into the cerebral cortex of mice with experimental intracerebral haemorrhage (ICH), compared to control NPCs [162].

Galectin (Gal)-1 is expressed in the adult brain by NPCs of the SVZ [163] and hippocampus [164]. This soluble lectin is also expressed around infarcted tissue after brain ischaemia and was found to inhibit the proliferation of astrocytes in a dose-dependent manner, attenuate astrogliosis and down-regulate the astrogliosis-associated expression of iNOS and IL-1β after brain ischaemia [165].

Following these observations, human NPC-Gal-1 were generated and transplanted into rodents with experimental unilateral focal brain ischaemia, thus reducing the total ischaemic volume and improving the recovery of motor deficits, compared with untransduced control NPCs [165–168].

NPCs overexpressing IL-10, an anti-inflammatory cytokine that efficiently suppresses EAE [169], showed an enhanced ability to promote remyelination, neuronal repair and immune suppression (both in the spinal cord and in the CNS), compared with untransduced NPCs, when systemically injected into EAE mice [170].

Many CNS diseases are due to the lack of a crucial enzyme, such as β-galactocerebrosidase (GALC) in the pediatric leukodystrophies[171]. Mouse NPCs, either untransduced or genetically modified to express supranormal levels of GALC, were transplanted directly into the brain of twitcher mice. NSC rapidly engrafted, migrated and stably expressed GALC *in vivo*, which widely distributed through the cerebrospinal fluid (CSF) and reached the whole brain and the spinal cord. Supraphysiological levels of GALC in donor cells resulted in enhanced metabolic reconstitution of host CNS tissue, which was mediated by cross-correction, the mechanism by which secreted lysosomal enzymes are uptaken by neighbouring cells. The levels of enzymatic reconstitution/correction correlated well with the reduction of tissue storage, decrease of activated astroglia and microglia, modulation of inflammation, and improved survival [171].

Thus, besides the secretion of cytokines and growth factors, the therapeutic effects of NPCs have been ascribed to the release of therapeutic enzymes that are taken up by cross-correction mechanisms to enzyme-deficient host brain cells. In this perspective, the genetic manipulation of NPCs applied to metabolic correction via secreted enzymes is another way to exploit the *SCS* potential and becomes crucial to counteract/prevent irreversible CNS damage.

As the biology mediating the therapeutic benefit of the MSC and NPC secretome becomes more defined, targeted *SCS* preconditioning and genetic manipulation approaches will likely be useful in enhancing therapeutic relevance.

## 6. Conclusions and future perspectives

Based on the encouraging pre-clinical results [172,173], a new paradigm has been proposed concerning the ability of implanted MSCs and NPCs to foster restorative endogenous responses via bioactive molecules secreted at the level of the injured tissue microenvironment.

The delivery into the CNS of single cytokines identified to be active in the *SCS* (e.g. the neuropoietic cytokine LIF), should in principle stimulate the proliferation of CNS-resident progenitor cells and increase its reparative potential; this is a strong premise for the treatment of disorders such as MS and SCIs in which acute *vs* chronic tissue damage leads to accumulating and irreversible disability [174].

However, the outcomes from early clinical trials with single cytokines for brain or cardiovascular diseases have failed to meet expectations [175,176]. The brain injection of HGF [177,178] has been tested *in vivo* and can be seen as the first attempt with a single molecule to harness the therapeutic potential of the *SCS*.

The intracerebroventricular [179] or putaminal [180,181] infusion of GDNF has recently shown to be an effective treatment for PD, with clinical trials currently in progress [182]. Trials with NGF for AD are ongoing, with the growth factor either given as infused protein [183], released *in situ* by implanted NGF-secreting cells [184] or adeno-associated gene delivery [185]. Preclinical data with BDNF also show much promise, yet there are accompanying difficulties mainly due to the short serum half-life, low distribution rate and poor access to the CNS by the systemically-injected protein [182].

The safety, tolerability and efficacy of single cytokine/growth factor therapies are matter of great debate in these clinical trials and the clarification of these issues will set the stage to think on the biosafety of the *SCS*, as plausible alternative. In fact, stem cells do not just secrete growth factors and/or cytokines, but instead release a wide range of products that can encourage the growth and even supplement (host) cells [141,186]. Multiple cytokines/growth factors may need to be administered simultaneously at different concentrations and time points to act synergistically to achieve a therapeutic effect. As such, the application of *SCS* in regenerative medicine is now opening new frontiers in the treatment of a potentially long list of neurological disorders [187], the characterization of the *SCS* being one of the main challenges of next-generation ‘-*omics*’ applied to stem cells and regenerative neuroscience.

Some practical considerations for harnessing the *SCS* in experimental and clinical settings need to be posed.

*First*, the limitations of cytokine-based therapy are mostly due to tissue transport, pharmacokinetics and protein stability, and this reinforces the need to develop controlled release and delivery strategies for the *SCS*. While the use of preconditioned media eliminates the need for direct injection of cells, the coupling of preconditioned media with bioengineered materials may increase the effect of these cytokines and chemokines and possibly extend the duration of their therapeutic effects. Substantial progress in the development of novel nanofibres for the delivery and controlled release of bioactive molecules has been made in recent years [188]. Wang and co-workers provided the first example of the effect of preconditioned nanofibres with ES cells for the recovery from lipopolysaccharide (LPS)-induced acute kidney injury *in vivo* [189]. Similarly encouraging results have been obtained with nanofibre hydrogels that act as sponges, thus soaking up the *SCS* while being separated from the stem cells themselves via a permeable membrane [190]. During incubation, the peptide gel is not in contact with the cells, but still permeable enough for the *SCS* to diffuse through the culture medium and into the gel. After this preconditioning step, the hydrogel can be used in a cell-free manner to treat the condition of interest [190].

A *second* relevant issue is the identification and characterization of stem cell EV cargoes and their exploitation as a secretome therapeutic component and natural delivery system that remain elusive. Alvarez-Erviti succeeded in delivering functional siRNA to the mouse brain by systemically injecting targeted exosomes [191]. This was the first demonstration of a naturally occurring exosome-based drug delivery system focused on specific secretome components. Based on the fact that EVs have similar beneficial effects in regenerative therapy to donor cells [112,125,192], producing EVs from stem cells could be envisaged, holding promising therapeutic relevance in brain repair on a large scale and even modifying their composition. This could be achieved by *in vitro* manipulation of stem cells to enhance secretion of pro-regenerative factors and the development of novel therapeutic strategies by using *in vitro* generated and modified MVs enriched with growth factors, cytokines, chemokines and regulatory miRNAs that promote regeneration of damaged tissue [114].

A *third* important aspect to consider is the patient’s safety and the potential benefit-to-risk ratio of stem cell therapies. A number of MSC- or NPC-based phase I and II clinical trials are being conducted [193–196], and there is great expectation that these pioneering studies will tell more about the biosafety as well as anticipate some efficacy outcomes of non-haematopoietic stem cell therapies for neural repair and/or restoration [193,194,197]. Furthermore, when prospecting the use of genetically engineered stem cells, or the manipulation of secreted EVs, the risk of tumour formation (or induction) due to insertional mutagenesis by integrating technologies still raises caution for human clinical applications [198].

*Fourth*, at least two main aspects of interest in the field of stem cells are being proposed by neuroethics (topics that neurosciences have put forth in dealing with traditional ethics): (i) those related to the origin of stem cells (e.g. from aborted foetuses for NPCs) and the way in which they are obtained, studied, protected, and preserved; and (ii) those related to the application of stem cells in neuroscience, from feasibility to viability (e.g. limited in time over extensive culturing for MSCs), risk and benefit, the transplant process itself, complications, outcome, public health impact, and also potential deviations [199].

A *fifth* additional main aspect to consider, which applies well also to syngeneic stem cells exposed to an inflammatory environment [9], is the immunogenicity of the graft, namely its ability to provoke an immune response when facing the host immune system after transplantation [200]. Some of the limitations anticipated by these two latter issues should be overcome by induced pluripotent stem (iPS) or induced stem (iS) cells, which both provide an alternative source of functional, stably expandable [71,201], and therapeutically efficacious MSCs or NPCs for tissue repair [202,203].

Although candidate molecules are under investigation, further detailed studies are needed to carefully define the factors responsible for the *SCS*-mediated/instructed neuroprotective and regenerative properties, in order to best capitalize upon this type of therapy for the repair of neurodegenerative diseases. Elucidation of the molecular pathways mediating stem cell *SCS* expression is a crucial step towards improving our understanding of the secreted factor profile and its clinical utility [204]. Besides proteomic analysis, attempts are now being made using a next-generation metabolomics-driven approach coupled with system biology techniques to clarify the key metabolic pathways mediating *SCS* expression. Elucidation of these mechanisms may be a crucial and essential step in the control and improvement of *SCS* to maximize therapeutic strategy in regenerative medicine.

Whereas determining the mechanism regulating the expression of the secretome is important, the task is made more challenging given that some of the proteins are released during cell death [127]. Therefore, the most important goal will be to move towards methods to profile the *SCS in vivo* that can distinguish between factors released from the host versus those secreted by the transplanted stem cells that can be dynamically regulated by the local microenvironment and harnessed in the *SCS*-based therapeutics. Reaching this point will require the development of new techniques that can directly quantify the dynamic expression profile of stem cells secreted factors both locally and systemically.

**Fig. 1:**
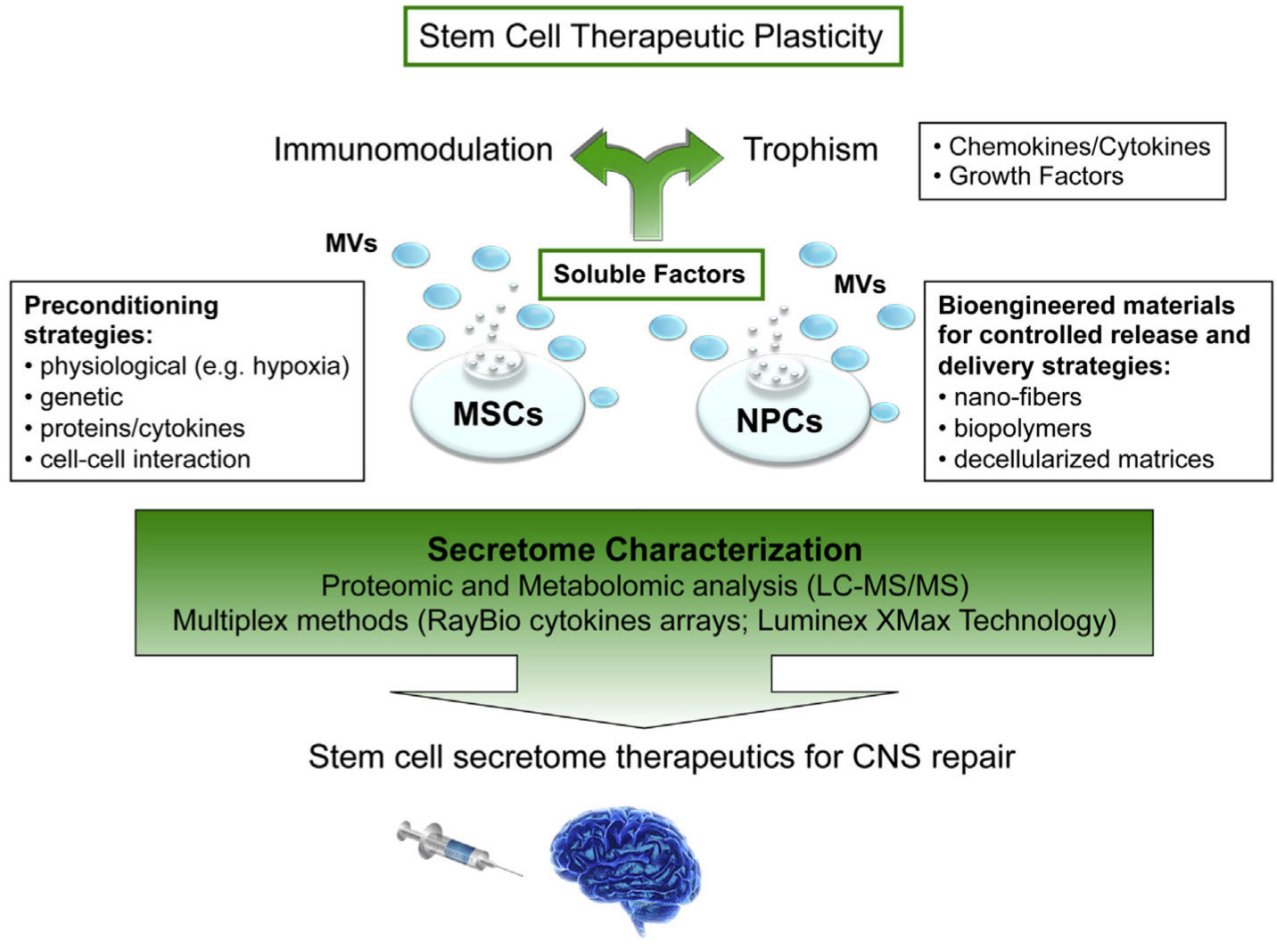
Proposed model for the therapeutic application of mesenchymal/neural stem cell secretome in CNS repair. Exchange of signals between grafted MSCs or NPCs and the host lead to remarkable tissue trophic effects on endogenous brain cells and beneficial modulatory actions on innate and adaptive immune responses, which ultimately promote the healing of the injured CNS. The characterization of the *SCS* is one of the main challenges of proteomics and metabolomics applied to neuroscience. Coupling of preconditioned media with bioengineered materials may be employed to control and sustain the expression of customized secretome and possibly extend the duration of the observed therapeutic effects.

In conclusion, beyond the great enthusiasm for new treatment perspectives, much investigative work is still in progress on the development of robust and customized SCS-based future therapeutics, paving the way for new regenerative medicine approaches to CNS repair (Fig. 1).
